# Preparation and Identification of ACE Inhibitory Peptides from the Marine Macroalga *Ulva intestinalis*

**DOI:** 10.3390/md17030179

**Published:** 2019-03-19

**Authors:** Siqi Sun, Xiaoting Xu, Xue Sun, Xiaoqian Zhang, Xinping Chen, Nianjun Xu

**Affiliations:** 1 School of Marine Sciences, Ningbo University, Ningbo 315211, China; 15728041077@163.com (S.S.); m17751232933@163.com (X.X.); sunxue@nbu.edu.cn (X.S.); zhangxiaoqian@nbu.edu.cn (X.Z.); 2 Division of Allergy, Pulmonary, and Critical Care Medicine, Vanderbilt University Medical Center, Nashville, TN 37232, USA; Xinping.chen@vanderbilt.edu

**Keywords:** *Ulva intestinalis*, ACE inhibitory peptide, optimization, purification, structural identification, molecular docking

## Abstract

Angiotensin I-converting enzyme (ACE) inhibitory peptides derived from seaweed represent a potential source of new antihypertensive. The aim of this study was to isolate and purify ACE inhibitory peptides (ACEIPs) from the protein hydrolysate of the marine macroalga *Ulva intestinalis*. *U. intestinalis* protein was hydrolyzed by five different proteases (trypsin, pepsin, papain, α-chymotrypsin, alcalase) to prepare peptides; compared with other hydrolysates, the trypsin hydrolysates exhibited the highest ACE inhibitory activity. The hydrolysis conditions were further optimized by response surface methodology (RSM), and the optimum conditions were as follows: pH 8.4, temperature 28.5 °C, enzyme/protein ratio (E/S) 4.0%, substrate concentration 15 mg/mL, and enzymolysis time 5.0 h. After fractionation and purification by ultrafiltration, gel exclusion chromatography and reverse-phase high-performance liquid chromatography, two novel purified ACE inhibitors with IC_50_ values of 219.35 μM (0.183 mg/mL) and 236.85 μM (0.179 mg/mL) were obtained. The molecular mass and amino acid sequence of the ACE inhibitory peptides were identified as Phe-Gly-Met-Pro-Leu-Asp-Arg (FGMPLDR; MW 834.41 Da) and Met-Glu-Leu-Val-Leu-Arg (MELVLR; MW 759.43 Da) by ultra-performance liquid chromatography-tandem mass spectrometry. A molecular docking study revealed that the ACE inhibitory activities of the peptides were mainly attributable to the hydrogen bond and Zn(II) interactions between the peptides and ACE. The results of this study provide a theoretical basis for the high-valued application of *U. intestinalis* and the development of food-derived ACE inhibitory peptides.

## 1. Introduction

Hypertension, a common, serious chronic disease, affects approximately 25% of the adult population worldwide. Hypertension seriously affects human health and is a causative factor of cardiovascular diseases, stroke, and renal diseases, among others [1,2]. The renin–angiotensin system (RAS) and kallikrein–kinin system (KKS) are crucial for regulating blood pressure in the human body. Angiotensin I-converting enzyme (E.C.3.4.15.1, ACE), a peptidase belonging to the zinc metalloenzyme family, plays an important role in RAS and KKS, inactivating angiotensin I to the potent vasoconstrictor angiotensin II and also inactivating the vasodilator bradykinin to raise blood pressure [3]. Therefore, inhibition of ACE activity is effective for maintaining blood pressure within a normal range [4].

ACE inhibitors (ACEIs) inhibit ACE activity and reduce blood pressure by inhibiting the synthesis of angiotensin II or promoting the release of bradykinin. Although ACEIs, such as enalapril, captopril, and lisinopril, are widely used in hypertension treatments, synthetic ACEIs have a series of negative effects, including hypotension, cough, increased potassium levels and, angioedema [5,6]. Consequently, the development of safe and effective antihypertensive drugs is important, and due to antihypertensive effects and safety, there has been an increasing interest in food-derived ACE inhibitory peptides (ACEIPs) during the last decades. To date, ACEIPs derived from a variety of products such as milk [7], bovine collagen [8], mushrooms [9], rice [10], and marine sources including fish, shellfish, and macroalgae [11,12], have been reported. Indeed, marine organisms, which are rich in unique bioactive compounds, are valuable for human health. Hence, there is much research attention in isolating bioactive compounds from marine organisms to develop new drugs or health products. Macroalgae are important bio-resource organisms in marine ecosystems. According to previous studies, many unique bioactive compounds, including peptides, fats, and carbohydrates, have been isolated from macroalgae [13,14]. Moreover, some novel ACE inhibitory peptides with efficient antihypertensive effects have been isolated from enzymatic hydrolysates of algal species. For instance, Cao et al. reported a peptide with an IC_50_ value of 474.36 μM (Gln-Val-Glu-Tyr) from hydrolyzed *Gracilariopsis lemaneiformis* [12]. Other peptides, such as Ala-Ile-Tyr-Lys (IC_50_ = 213 μM), Tyr-Lys-Tyr-Tyr (IC_50_ = 64.2 μM), Lys-Phe-Tyr-Gly (IC_50_ = 90.5 μM), and Tyr-Asn-Lys-Leu (IC_50_ = 21 μM) from *Undaria pinnatifida* [15] and Ile-Tyr (IC_50_ = 2.69 μM), Ala-Lys-Tyr-Ser-Tyr (IC_50_ = 1.52 μM), Leu-Arg-Tyr (IC_50_ = 5.06 μM), and Met-Lys-Tyr (IC_50_ = 7.26 μM) from *Porphyra yezoensis* [16] have been found. In addition, two peptides (Ile-Pro and Ala-Phe-Leu) with IC_50_ values of 87.6 μM and 65.8 μM were purified from an *Ulva rigida* protein hydrolysate [17]. Some peptides have exhibited powerful antihypertensive effects comparable to those of pharmaceutical drugs in spontaneously hypertensive rats (SHRs) [16,18]. Thus, marine algae can be used as a new source of ACEIPs.

*Ulva intestinalis*, a marine green algae belonging to the family of Ulvaceae, consisting of a tubular frond and unbranched thalli [19]. It is able to reproduce using unfused gametes, spores, and zygotes. Under suitable growth conditions *U. intestinalis* can quickly occupy the littoral zone [20], and it is among the species that cause green tides, which can affect the growth of other coastal organisms [21,22]. In addition, *U. intestinalis* is regularly consumed in the East Asian countries of China, Korea, and Japan. It has been reported that *U. intestinalis* is rich in vitamins (0.174 mg/g), proteins (~20.5%), carbohydrates (42.1%), and other bioactive compounds [23,24], and the high content of crude proteins in *U. intestinalis* renders it a potential source of ACE inhibitory peptides for the functional foods and medical industries. To the best of our knowledge, no study to date has aimed at purifying and characterizing ACE inhibitory peptides from *U. intestinalis*.

In this study, *U. intestinalis* was hydrolyzed using five different proteases (trypsin, pepsin, papain, α-chymotrypsin, alcalase), and response surface methodology (RSM) was employed to optimize the hydrolysis conditions, including pH, hydrolysis temperature, substrate concentration, and enzyme/substrate ratio (E/S). The hydrolysate solution was fractionated using nominal molecular weight limit (NMWL) Amicon Ultra-15 centrifugal filters, and bioactive peptides were further purified and identified using Sephadex G-25, G-15 gel chromatography, reverse-phase high-performance liquid chromatography (RP-HPLC), and ultra-performance liquid chromatography-tandem mass spectrometry (UPLC-MS/MS). Furthermore, the peptides were chemically synthesized and then used for the determination of their stability during gastrointestinal digestion.

## 2. Results and Discussion

### 2.1. Preparation of ACE Inhibitory Peptides from U. intestinalis

**Figure 1:**
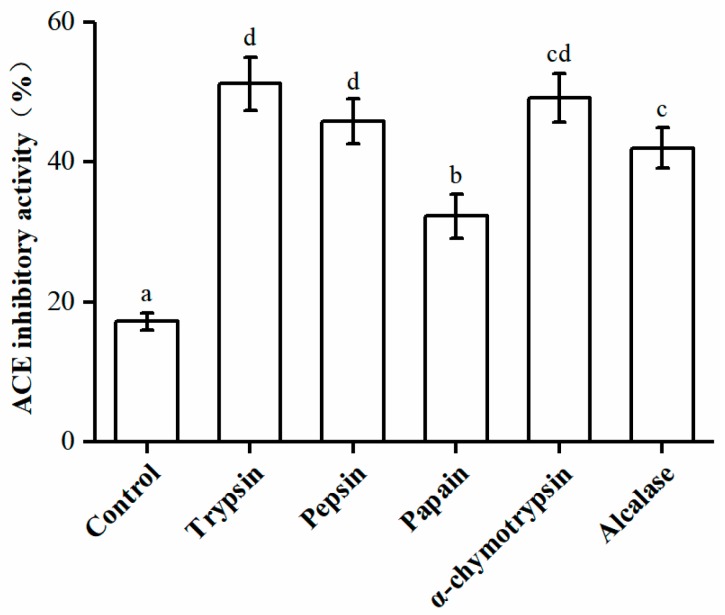
Angiotensin I-converting enzyme (ACE) inhibitory activities of *U. intestinalis* protein hydrolysates produced by different enzymes. Each point is the mean of three determinations (*n* = 3) ± SD. Different letters indicate significant differences. The concentration of each hydrolysate was 2.5 mg/mL; crude protein (2.5 mg/mL) was used as the positive control.

Proteases are necessary to release ACEIPs from inactive forms [25]. Different proteases affect the composition and size of the polypeptides produced, which can affect their biological activities [26]. In this study, *U. intestinalis* proteins were hydrolyzed using five different proteases, and their ACE inhibitory activities were assessed (Figure 1). The trypsin-hydrolyzed product showed the greatest ACE inhibitory activity (51.15 ± 3.78%). Thus, trypsin was chosen for the production of ACEIPs. 

**Figure 2:**
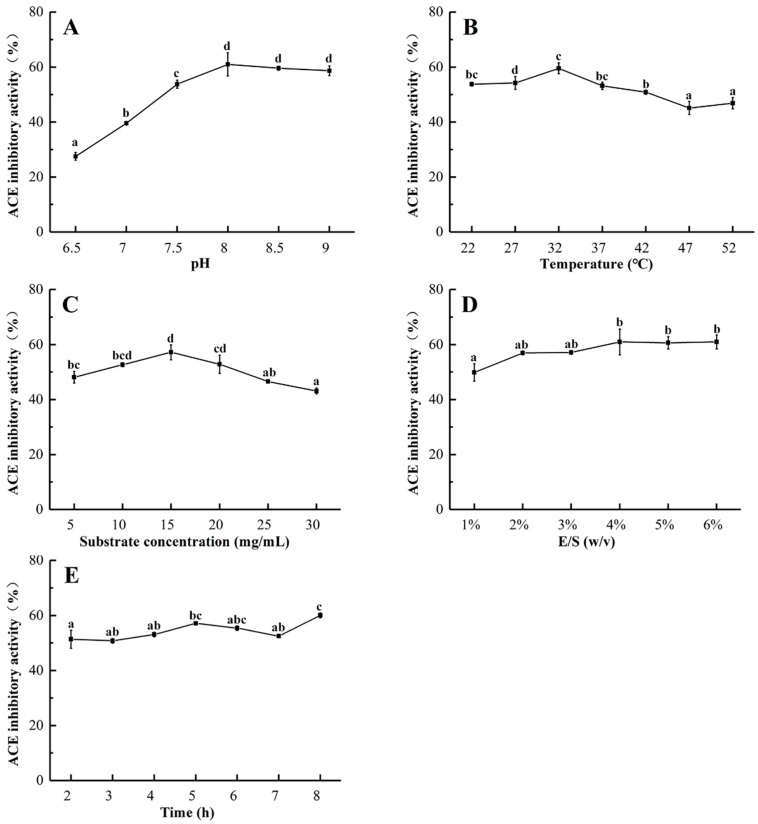
Effects of hydrolysis pH (**A**), temperature (**B**), substrate concentration (**C**), E/S ratio (**D**), and reaction time (**E**) on ACE inhibitory activity of protein hydrolysates from *U. intestinalis*.

Various factors such as temperature, pH, and substrate concentration affect protein extraction from marine sources [27], and the effect of pH on the ACE inhibition rate of the hydrolysates was significant, as shown in Figure 2A. With a rise in pH, the ACE inhibitory activity of the protein hydrolysates increased to a maximum value (60.98%) at pH 8.0; there was no significant difference in ACE inhibitory activity at a higher pH. A certain range of pH can affect the degree of dissociation of enzyme molecules and substrates, and promote the binding of enzymes to substrates. Therefore, the primary enzyme solution pH is 8.0.

Temperature is also an important factor for influencing enzyme activity and hydrolysis efficiency. Figure 2B shows the effect of temperature on the ACE inhibitory activity of protein hydrolysates. ACE inhibitory activity gradually increased from 22 to 32 °C, and the maximum value was reached at 32 °C (59.53%), above which the inhibitory activity decreased. The reason for this may be that trypsin is gradually activated as the temperature of enzymatic hydrolysis increases, resulting in more active peptide fragments, and the structure of the enzyme protein was affected when the temperature exceeded the optimum temperature. Therefore, the optimal temperature for the production of ACEIPs from *U. intestinalis* was determined to be 32 °C (Figure 2B).

The effect of substrate concentration from 5 to 30 mg/mL on the ACE inhibitory activity of *U. intestinalis* protein hydrolysates was also evaluated. As presented in Figure 2C, inhibitory activity increased from 5 to 10 mg/mL, but rapidly decreased from 15 to 30 mg/mL. This may have been due to the high concentration of substrate affecting the binding of the protease to the substrate, thereby inhibiting the enzymatic reaction. The highest value of ACE inhibitory activity (57.19%) was observed at a substrate concentration of 15 mg/mL, which was chosen for further experiments.

The effect of E/S on the ACE inhibitory activity of the hydrolysates was also studied. At a range from 1 to 6%, the ACE inhibitory activity reached a maximum (60.94%) at an E/S of 4% (Figure 2D). Thus, 4%was considered to be the optimal E/S in this study. The possible explanation for this is that an increase in the E/S, which increases the chance of contact between the substrate protein and the enzyme, accelerates the enzymatic reaction and produces more active peptides. 

As depicted in Figure 2E, no significant difference in ACE inhibitory activity was observed when the reaction time increased from 2 to 8 h. In the interest of time concerns, 2 h was chosen as the hydrolysis time for ensuing experiments.

### 2.2. Optimization of the Enzymatic Hydrolysis Condition

RSM was utilized to optimize the enzymatic hydrolysis conditions with regard to three significant factors for the production of ACE inhibitory peptides, including pH (*X*1), temperature (*X*2), and substrate concentration (*X*3). The factors and levels are provided in Table 1. The experimental design and Box–Behnken results for the incubation conditions are shown in Table 2, where *Y* represents the ACE inhibitory activity and *X*1, *X*2, and *X*3 represent the pH, temperature, and substrate concentration, respectively.

The data were analyzed to obtain a quadratic regression model using Design Export 8.0.6 (StatEase, Inc, USA). A multiple regression equation correlating to the response function with independent variables was as follows:

*Y* = 62.70 + 3.01A − 0.62B + 0.25C − 3.19AB + 2.54AC + 0.072BC − 4.57A^2^ − 2.33B^2^ − 0.59C^2^ (1)

The results of analysis of variance and the fitness of the model are summarized in Table 3. Overall, pH, pH and temperature, and pH and substrate concentration had significant effects on inhibitory activities (*p* < 0.001). As the value of the “lack of fit” item was *p* = 0.5449, unknown factors had little influence on the results. 

As indicated in Table 3, the regression model was also used to fit the effect of three factors on the ACE inhibition rate. The coefficient of multiple determinations (*R*^2^) for the quadratic regression model was 0.9808; thus, a 98.08% response to the ACE inhibition rate was caused by the concentration of A, B, and C and their interactions. Moreover, the value of adjusted determination coefficients (*R*^2^adj) and *R*^2^ were both close to 1 (*R*^2^ = 0.9808 and *R*^2^_adj_ = 0.9560), indicating that this model may be used to analyze and predict changes in ACE inhibitory activity under different enzymatic hydrolysis conditions [28].

**Figure 3:**
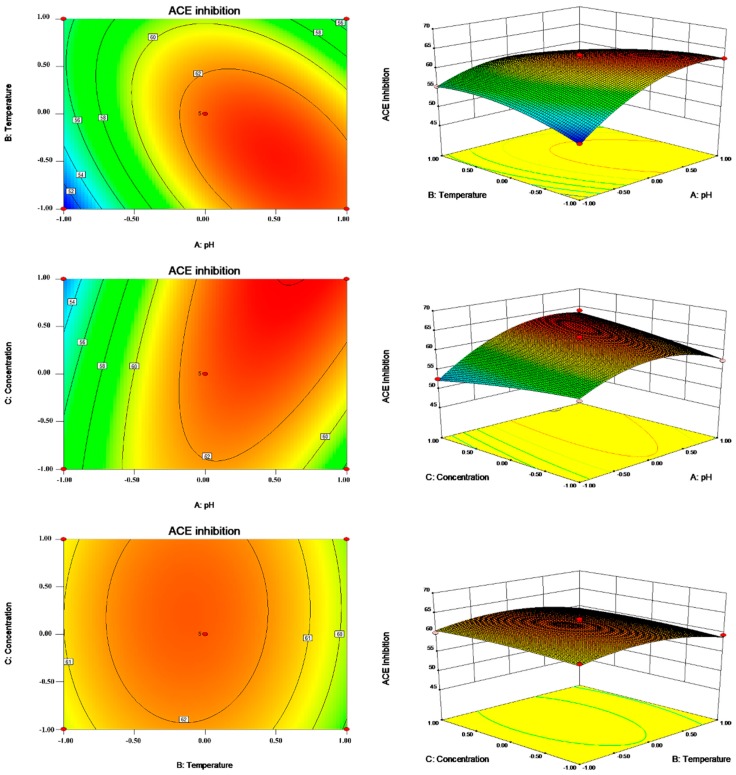
Contour plots and response surface plots for pH (**A**), temperature (**B**), and substrate concentration (**C**) to ACE inhibition rate.

In our present study, response surface plots and contour plots were applied to demonstrate the effect and interaction of independent variables on the ACE inhibitory rates of protein hydrolysates. Figure 3 illustrates the effect of X1 and X2 on such ACE inhibitory activities.

The optimum condition for ACE inhibitory activity was obtained at pH 8.42, 28.5 °C, a substrate concentration of 15 mg/mL, an E/S of 4%, and an enzymolysis time of 5 h. Under optimal reaction conditions, the predicted and experimental values for the ACE inhibitory activities of the protein hydrolysates were 64.91 and 64.07%, respectively, indicating that the predicted value was close to the experimental value. Thus, the parameters obtained by the RSM optimizations were reliable, and it is feasible to use them in practice.

### 2.3. Purification of ACE Inhibitory Peptides

Ultrafiltration can separate protein hydrolysates into components of different molecular weights (MWs), and active peptides with different components have different biological activities. It has been reported that peptides with molecular weights <3 kDa generally possess high ACE inhibitory activity [29,30]. In the present study, the hydrolysate was separated into three fractions (<3 kDa, 3–10 kDa, and >10 kDa) by filtering with ultrafiltration membranes. The IC_50_ value and ACE inhibitory activity at 1.5 mg/mL of each fraction were assessed, and the results are displayed in Table 4. Among the fractions, the MW < 3 kDa fraction exhibited the strongest ACE inhibitory activity, with an inhibitory rate of 53.01%. In contrast, peptides with a high molecular weight (MW > 10 kDa) showed lower ACE inhibitory activity (Table 4). Thus, the fraction with MW < 3 kDa was chosen for further separation and purification of ACEIPs. 

**Figure 4:**
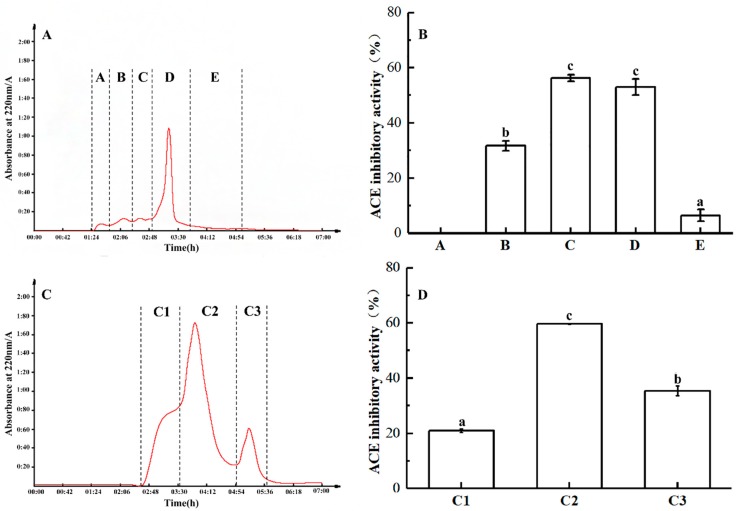
Sephadex G-25 gel filtration chromatogram of <3 kDa fraction of trypsin hydrolysate from *U. intestinalis.* (**A**) The fraction was divided into five parts (**A**–**E**) by Sephadex G-25. (**B**) The ACE inhibitory activity (1.5 mg/mL) and percentage of **A**–**E**. (**C**) The fraction was divided into three parts (C1–C3) by Sephadex G-15. (**D**) The ACE inhibitory activity (1.5 mg/mL) and percentage of C1 to C3.

The MW < 3 kDa fraction was further separated using a Sephadex G-25 gel filtration column, with five major peaks at 220 nm (Figure 4A): A, B, C, D, and E. At a concentration of 1.5 mg/mL, fractions B, C, and D showed inhibitory activity against ACE, with minimal activity for fraction A. With an inhibitory rate of 56.3%, fraction C exhibited the greatest ACE inhibitory activity (Figure 4B).

Accordingly, fraction C was further separated using a Sephadex G-15 gel filtration column, revealing three major peaks (C1–3) at 220 nm (Figure 4C). Among them, fraction C2 showed the highest activity (Figure 4D). 

**Figure 5:**
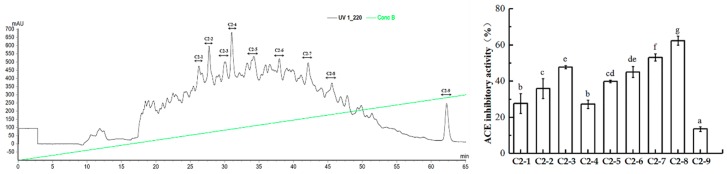
ACE inhibitory activity of fraction C2 from RP-HPLC. (**A**) The fraction was divided into 9 parts (C2-1 to C2-9) by RP-HPLC. (**B**) ACE inhibitory activity (1.5 mg/mL) and percentages of C2-1 to C2-9. Means with different lower case letters are significantly different (*p* < 0.05).

Fraction C2 was then concentrated and used for further separation by means of RP-HPLC. The solution was purified on an AKTA pure system (GE Healthcare, Uppsala, Sweden) with an Inertsil ODS-3 C18 column (φ10 × 250 mm). Nine peaks were collected separately (Figure 5A). Among those fractions, C2-8 exhibited the most potent ACE inhibitory activity, with an inhibitory rate of 62.35% (Figure 5B). Then, the fraction C2-8 was further purified by HPLC.

**Figure 6:**
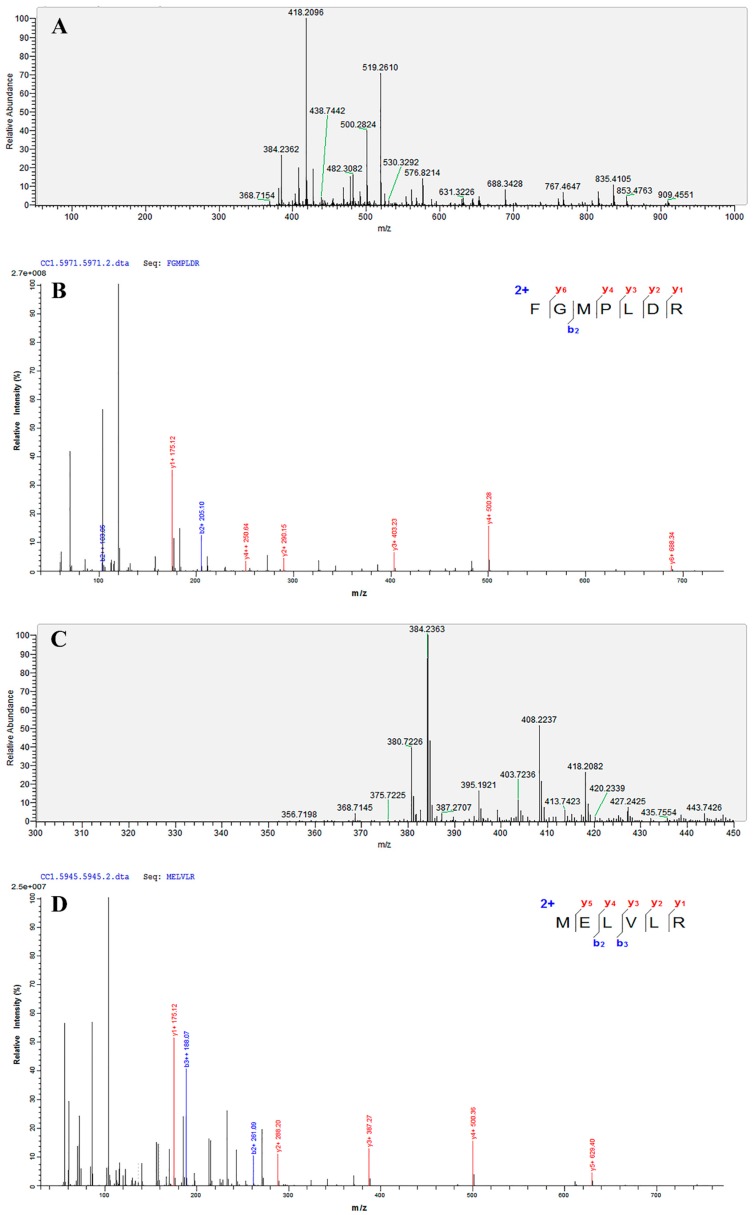
The primary mass spectrogram and corresponding secondary mass spectrogram of FGMPLDR and MELVLR. (**A**) MS/MS spectra of FGMPLDR. (**B**) The secondary mass spectrogram of FGMPLDR. (**C**) MS/MS spectra of MELVLR. (**D**) The secondary mass spectrogram of MELVLR.

As shown in Table 5, two ACEIPs were obtained from fraction C2-8 by UPLC-MS/MS and de novo sequencing. According to mass spectra determined by UPLC-MS/MS (Figure 6), Mascot software identified Phe-Gly-Met-Pro-Leu-Asp-Arg and Met-Glu-Leu-Val-Leu-Arg (FGMPLDR and MELVLR; Matrix Science, Inc, USA), which are novel peptides with ACE inhibitory activity from *U. intestinalis* activity not previously reported. The IC_50_ values of FGMPLDR and MELVLR were 219.35 μM and 236.85 μM, respectively. Previous studies have shown that the amino acid composition of a peptide has a significant effect on its ACE inhibitory activity [31], and it has been reported that ACE prefers to bind to a polypeptide with a high content of hydrophobic amino acids [32]. The two peptides obtained in this experiment both have a hydrophobic amino acid content, 42.9 and 50%, for FGMPLDR and MELVLR, respectively, which may contribute to their high activities. Furthermore, FGMPLDR and MELVLR are composed of 6–7 amino acids, which may also play a crucial role in their ACE inhibitory activities. According to previous studies, most ACEIPs are small peptides of 2–12 residues and molecular weights less than 3000 Da [33], which may more easily fit in the ACE active site and thus assert inhibitory activity [34]. The composition of the C- and N-terminal residues of an ACEIP also have a significant effect on ACE inhibition rate, with high activity when the C-terminal residue is Tyr, Phe, Pro, Trp, or Leu and the N-terminal residue is a hydrophobic aliphatic branched-chain amino acid such as Leu, Ile, Ala, or Met. Moreover, positively charged amino acids such as Arg and Lys at the C-terminus and a basic amino acid (Arg, Lys, and His) at the N-terminus can enhance the affinity of the peptide for ACE, further increasing antihypertensive activity [35,36,37]. The C-terminal amino acids of the two peptides in our study (FGMPLDR and MELVLR) were Arg, which was consistent with the results of a previous study [36].

### 2.4. In Vitro Stability of ACEIPs Derived from U. intestinalis

After gastrointestinal digestion, some food-derived ACEIPs do not exhibit (or exhibit fewer than) the expected hypotensive effects [38,39]. Thus, to evaluate resistance to gastrointestinal enzymes, the ACEIPs obtained in our study were subjected to a two-step hydrolysis process. After digestion with pepsin and trypsin, the ACE inhibitory activities of FGMPLDR and MELVLR were 51.32 and 58.63%, respectively, with no significant difference from the control (Table 6). Therefore, our results indicate that these peptides are stable in the gastrointestinal tract and may also show effective antihypertensive activity in vivo.

### 2.5. Molecular Docking

**Figure 7:**
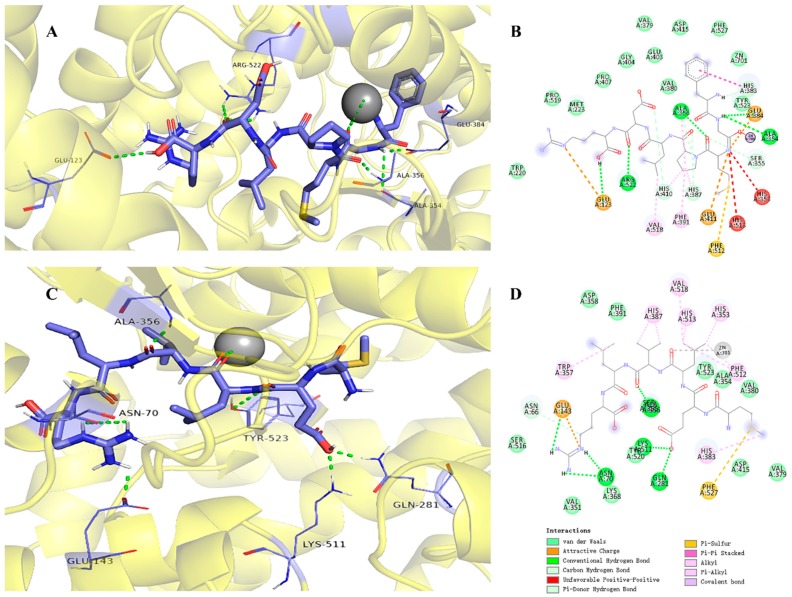
Molecular docking results for FGMPLDR and MELVLR with ACE (PDB: 1O8A). (**A**) 3-D details of ACE and FGMPLDR interactions. (**B**) 2-D interaction details for FGMPLDR. (**C**) 3-D details of ACE and MELVLR interactions. (**D**) 2D interaction details for MELVLR.

To elucidate the inhibitory mechanism, docking simulation was conducted using AutoDock 4.2 software. The best results were obtained for FGMPLDR and MELVLR at the ACE active site in the presence of Zn(II) (Figure 7), with binding energies of −2.78 kcal/mol and −6.04 kcal/mol, respectively. This is a reference for assessing the binding between proteins and peptides [38,40]. The peptides and ACE residues are mainly linked through hydrogen bonds, hydrophobic interaction, and polar, Van der Waals, and electrostatic forces. It has been reported that hydrogen bond interactions play an irreplaceable role in stabilizing the structure of the complex as well as the ACE reaction [41,42]. Previous studies have indicated three main active site pockets in the ACE molecule. The S1 pocket includes three residues, Ala354, Glu384, and Tyr523, and the S2 pocket Gln281, His353, Lys511, His513, and Tyr520; in contrast, the S1′ pocket only includes residue Glu162 [43]. Furthermore, the lisinopril, an ACE inhibitor, was found to share interactions at Ala354, His383, Glu384, and Lys511, showing that those residues might play major roles in ACE binding [44,45]. Our molecular docking studies indicated that FGMPLDR and MELVLR bind to the active site pocket of ACE through a network of hydrogen bonds and hydrophobic and Van der Waals interactions. Both peptides displayed a stable docking structure with ACE. As shown in Figure 7B, five hydrogen bonds between FGMPLDR and residues Glu123, Ala354, Ala356, Glu384, and Arg522 of ACE were formed, and the Van der Waals forces for nine residues were also important. Namely, two hydrogen bonds with the S1 pocket (Ala354 and Glu384) were produced for FGMPLDR. In the case of MELVLR, it formed six hydrogen bonds with residues Asn70, Glu143, Gln281, His383, and Lys511, and hydrophobic interactions with seven residues (Figure 7D). Gln281 and Lys511 of the S2 pocket associated with MELVLR through two hydrogen bonds. Furthermore, as ACE is a metalloenzyme with a zinc ion coordinated in the active site with His348, Glu372, and His344 [38], the presence of Zn(II) plays an important role in ACE inhibition [46]. For peptides FGMPLDR and MELVLR, Gly and Leu are coordinated to the Zn(II) ion, respectively. This interaction may have caused distortion of the tetrahedrally coordinated Zn(II), which further resulted in the loss of ACE activity.

## 3. Materials and Methods

### 3.1. Materials and Chemicals

Fresh *U. intestinalis* was collected from *Porphyra yezoensis* aquaculture rafts (N 29°44′, S 121°54′). The samples were washed with sterile water twice to remove any adherents and necrotic parts and then dried on paper. 

Trypsin, pepsin, papain, α-chymotrypsin, alcalase, and 3.5-kDa dialysis tubing were purchased from Solarbio Science and Technology Co., Ltd. (Beijing, China). ACE (from rabbit lung), *N*-[3-(2-Furyl)acryloyl]-Phe-Gly-Gly (FAPGG), Sephadex G-15, Sephadex G-25, and acetonitrile (ACN) were obtained from Sigma Chemical Co. (St. Louis, MO, USA). The 10-kDa and 3-kDa nominal molecular weight limit (NMWL) Amicon Ultra-15 centrifugal filters were purchased from Merck Millipore (Darmstadt, Germany). Other chemicals and reagents were of analytical grade.

### 3.2. Preparation of U. intestinalis Protein

Freeze-thaw with the frequency ultrasonic method [47] was used to extract protein from *U. intestinalis*. Samples were ground with three volumes of 2% NaCl. The mixture was frozen at −40 °C for 6 h and then incubated in a water bath at 20 °C to thaw (repeated three times). The mixture was further extracted using an ultrasonic processor (Scientz-ⅡD; Scientz, Ningbo, China) at 25 kHz at 300 W for 25 min, and the homogenates were centrifuged (Sorvall ST 16R Centrifuge, Thermo Electron LED GmbH, Osterode, Germany) with 8000 rpm for 15 min at 4 °C. Ammonium sulfate was added to the supernatant at a concentration of 60% to precipitate protein, which was collected by concentration (4 °C, 8000 rpm, 15 min). The protein pellet was dissolved in distilled water and dialyzed for 48 h at 4 °C using a 3.5-kDa MWCO dialysis bag against distilled water; the dialyzed retentate was lyophilized.

### 3.3. Enzymatic Hydrolysis of U. intestinalis Protein

*U. intestinalis* protein was digested with trypsin (37 °C, pH 8.0), pepsin (37 °C, pH 2.0), papain (37 °C, pH 6.0), α-chymotrypsin (37 °C, pH 8.0), and alcalase (37 °C, pH 10.0) for 5 h.The substrate concentration and enzyme/protein ratio were fixed at 20.0 mg/mL and 4% (*w*/*w*), respectively. The reaction was stopped by heating at 100 °C for 15 min, and the protein hydrolysates were centrifuged with 8000 rpm at 4 °C for 15 min. The supernatants were lyophilized and stored at −20 °C until use.

### 3.4. Single-Factor Experimental Design

Single-factor experiments were designed to obtain relevant factors for the production of ACEIPs and the experimental ranges of RSM. Based on previous experiments, trypsin was chosen as the optimal enzyme. The digestion conditions for the single-factor experiments, including pH (6.5, 7.0, 7.5, 8.0, 8.5, and 9.0), temperature (22, 27, 32, 37, 42, and 47 °C), substrate concentration (5.0, 10.0, 15.0, 20.0, 25.0, 30.0, and 35.0 mg/mL), E/S (1.0, 2.0, 3.0, 4.0, 5.0, and 6.0%), and reaction time (2.0, 3.0, 4.0, 5.0, 6.0, and 7.0 h), were investigated to reveal their influences on the ACE inhibitory activity of the protein hydrolysates.

### 3.5. RSM Experimental Design

In combination with the results obtained from the single-factor experiment, RSM was applied to optimize the enzymatic hydrolysis conditions. E/S and the reaction time were kept constant, at 4.0% and 5 h, respectively. The independent variables included pH (*X*1), temperature (*X*2), and substrate concentration (*X*3); the response variable (*Y*) was ACE inhibitory activity. The processing parameters were optimized using a Box–Behnken design, and each selected variable was coded at three levels (−1, 0, +1).

The software Design-Expert 8.0.6 was used to perform the experimental design and regression analysis of the experimental data. For verification of the predictive enzymatic hydrolysis conditions model, we further determined the ACE inhibitory activity of the enzymatic hydrolysate produced under optimum conditions.

### 3.6. ACE Inhibition and IC_50_ Assay

ACE inhibition was examined according to Shalaby et al. and Henda et al. [48,49], with slight modifications. FAPGG and ACE were dissolved in 50 mM Tris-HCl (pH 7.5) containing 300 mM NaCl. A sample solution (40 μL) was mixed with 100 μL of 0.88 mM FAPGG solution, and the mixture was incubated at 37 °C for 5 min. To start the reaction, 60 μL of ACE solution (0.20 U/mL) was added; absorbance was measured at 340 nm and recorded every 1 min for 30 min using a SpectraMax 190 absorbance microplate reader (Molecular Devices, Sunnyvale, USA). The slope averaged over a linear interval of 10–30 min was taken as a measure of the ACE inhibitory activity. The degree of ACE inhibition was calculated according to the following equation:

ACE inhibition rate (%) = [1 − (slope inhibitior/slope control)] × 100(2)

The activity of each sample was tested in triplicate. The IC_50_ value, the concentration of peptide required to reduce ACE activity by 50%, was determined by regression analysis of ACE inhibition (%) vs. peptide concentration.

### 3.7. Separation and Purification of ACE Inhibitory Peptide

#### 3.7.1. Ultrafiltration Separation

The hydrolysate solution was passed through ultrafiltration membranes with molecular weight (MW) cutoffs of 10 and 3 kDa to obtain three fractions (MW < 3 kDa; MW 3–10 kDa; MW > 10 kDa). Each fraction was assessed for ACE inhibitory activity and IC_50_.

#### 3.7.2. Gel Filtration Chromatography Analysis

The fraction with the highest ACE inhibition rate was applied to a Sephadex G-25 column (φ2.5 × 60 cm) at a flow rate of 0.5 mL/min using an MC99-3 automatic liquid chromatograph (Shanghai Huxi Analysis Instrument Factory Co., Ltd., Shanghai, China). Elution was measured at 220 nm, and 3-mL fractions were collected. Each major peak was pooled and lyophilized for ACE inhibitory activity assays. Fraction C with the highest activity was further fractionated using a Sephadex G-15 gel filtration column (φ1.0 × 60 cm) with the same operation as described above for Sephadex G-25. 

#### 3.7.3. RP-HPLC Analysis of ACE Inhibitory Peptides

The selected fraction C2 was fractionated using semi-preparatory RP-HPLC. Briefly, 7.5 mg of the peptide fraction was dissolved in 1 mL 0.05% TFA (*v*/*v*) and passed through a 0.2-μm filter. The solution was purified using an AKTA pure system (GE Healthcare, Uppsala, Sweden) with an Inertsil ODS-3 C18 column (φ10 × 250 mm). Ultra-pure water containing 0.1% TFA (*v*/*v*) was used as mobile phase A, and acetonitrile containing 0.1% TFA (*v*/*v*) was used as mobile phase B. Separation of the peptides was carried out at a flow rate of 1.5 mL/min using the following linear gradient: 0 to 50% eluent B from 0 to 65 min. Elution peaks were monitored at 220 nm. The purity of the fraction with the highest activity was further analyzed using a ZORBAX Eclipse XDB-C18 column (5 μm, φ4.6 mm × 150 mm; Agilent, USA). The column was eluted with a linear gradient of 0–35% mobile phase B from 0 to 25 min at a flow rate of 1 mL/min. Collected peaks were concentrated using a rotary evaporator system and lyophilized for later use.

### 3.8. Identification of the Amino Acid Sequence by UPLC-MS/MS

Identification of amino acid sequences was achieved using an Acquity UPLC system (Waters, USA) equipped with an Eksigent ChromXP C18 column (3 μm, φ250 nm × 75 μm). The mobile phase consisted of solvent A (0.1% formic acid in acetonitrile, *v*/*v*) and solvent B (0.1% formic acid in water, *v*/*v*), and the elution conditions were as follows: 0.0–45.0 min, 95–70% A; 45.0–52.0 min, 70–20% A; 52.0–53.0 min, 20–95% A; 53.0–60.0 min, 95% A. The flow rate was set at 300 nL/min. The injection volume was 4 μL.

A Q-Exactive Orbitrap mass spectrometry instrument (Thermo Fisher Scientific, USA) was employed for the identification and quantification of ACEIPs from *U. intestinalis.* The conditions were set as follows: full MS, resolution 70,000, AGC 1e6, scan range 350–1600 *m*/*z*; dd-MS2, resolution 17,500, AGC 2e5, isolation window 2.0 *m*/*z*. The amino acid sequences of the peptides were determined by de novo sequencing using PEAKS Studio 6. Using a solid-phase method, synthetic peptides (purity >95% by HPLC) were obtained from China Peptides Co., Ltd (Shanghai, China).

### 3.9. In Vitro Digestion

In vitro digestion was performed according to the method of Cing et.al [50]. The purified peptide sample was mixed with 0.1 M phosphate buffer (pH 2.0) and pepsin (E/S 4%, *w*/*w*); the reaction was allowed to proceed for 2 h at 37 °C and stopped by heating at 100 °C for 10 min. Subsequently, the remaining suspension was adjusted to pH 8.0 with 5 M NaOH solution and further digested with trypsin (E/S 4%, *w*/*w*); the solution was incubated at 37 °C for 2 h and then heated at 100 °C for 10 min. The protein hydrolysate was centrifuged at 8000 rpm/min for 15 min, and the supernatant was assayed for ACE inhibitory activity.

### 3.10. Molecular Docking

The structures of the peptides were constructed with minimal energy using Chem Office 15.1 software (CambridgeSoft Co., USA). The crystal structure of human ACE (PDB: 1O8A) was obtained from RCSB PDB (Protein Data Bank; http://www.rcsb.org/pdb/home/home.do). Water molecules and lisinopril were removed from the ACE model before docking, whereas the cofactor zinc and chloride atoms were retained in the ACE model. The flexible docking tool of AutoDock 4.2 software (TSRI, USA) was used for docking. The docking runs were carried out as follows: coordinates *x* 41.586, *y* 37.383, and *z* 43.445; grid box size of 90 × 90 × 90 Å. The best molecular docking was considered the output based on docking scores and the binding-energy value derived from the best poses of the peptides interacting with ACE.

### 3.11. Statistical Analyses

Statistical analyses were performed in triplicate for each sample, and the results are expressed as the mean ± standard deviation (SD). One-way analysis of variance (ANOVA) was used to determine differences between the mean values of triplicate groups. Statistical significance was established at *P* < 0.05 with the least significant difference (LSD) procedure of SPSS version 19.0.

## 4. Conclusions

In this study, *U. intestinalis* proteins were enzymatic hydrolyzed using five different proteases, with the trypsin-digested hydrolysates exhibiting the highest ACE inhibition rate. Based on single-factor analysis and the RSM method, the optimum conditions were as follows: pH 8.4, temperature 28.5 °C, E/S 4.0%, substrate concentration 15 mg/mL, enzymolysis time 5.0 h. After a series of chromatographic separation and purification steps, two novel ACE inhibitory peptides were identified: Phe-Gly-Met-Pro-Leu-Asp-Arg (FGMPLDR; MW 834.41 Da) and Met-Glu-Leu-Val-Leu-Arg (MELVLR; MW 759.43 Da). The purified peptides displayed potent ACE inhibitory activity, with IC_50_ values of 219.35 and 236.85 μM, respectively. Based on in vitro digestion results, FGMPLDR and MELVLR demonstrated good stability for pepsin and trypsin digestion. Furthermore, we investigated interactions between the peptides and ACE by molecular docking, and the results indicated that hydrogen bonds and interaction with the Zn^2+^ of ACE contribute to the ACE inhibition activities of the two peptides. Overall, these peptides derived from trypsin hydrolysates of *U. intestinalis* may be considered for use in the industrial production of functional foods. However, further research such as antihypertensive activity experiments in mice should be performed.

## Figures and Tables

**Table 1:** Coded values and independent variables of the response surface experiment.

Coded Level	Independent Variable		
	*X*1: pH	*X*2: Temperature (°C)	*X*3: Substrate Concentration (mg/mL)
−1	7.5	27	10
0	8.0	32	15
1	8.5	37	20

**Table 2:** Coded values and independent variables of the response surface experiment.

No	*X*1	*X*2	*X*3	ACE Inhibition (%)
1	−1	−1	0	50.43
2	1	−1	0	62.61
3	−1	1	0	55.36
4	1	1	0	54.78
5	−1	0	−1	56.23
6	1	0	−1	57.39
7	−1	0	1	52.61
8	1	0	1	63.91
9	0	−1	−1	60.58
10	0	1	−1	59.42
11	0	−1	1	60.00
12	0	1	1	59.13
13	0	0	0	61.74
14	0	0	0	61.74
15	0	0	0	63.04
16	0	0	0	63.48
17	0	0	0	63.48

**Table 3:** Variance analysis for ACE inhibitory activity in the RSM test.

Source	Sum of Squares	Df	Mean Square	*F* Value	*p* Value	Significance
Model	262.15	9	29.13	39.67	<0.0001	**
A-pH	72.35	1	72.35	98.53	<0.0001	**
B-Temperature	3.04	1	3.04	4.13	0.0815	
C-Substrate concentration	0.51	1	0.51	0.7	0.4301	
AB	40.66	1	40.66	55.38	0.0001	**
AC	25.73	1	25.73	35.04	0.0006	**
BC	0.021	1	0.021	0.029	0.8705	
A^2^	88.03	1	88.03	119.89	<0.0001	**
B^2^	22.78	1	22.78	31.03	0.0008	**
C^2^	1.45	1	1.45	1.98	0.2026	
Residual	5.14	7	0.73			
Lack of Fit	1.96	3	0.65	0.82	0.5449	
Pure Error	3.18	4	0.79			
Corrected Total	267.29	16				

Df: degrees of freedom, MS: mean square, *F* and *p* values, respectively ** *p* < 0.001, extremely significant.

**Table 4:** The ACE inhibitory of activity of the fraction separated by ultra-filtration.

Fraction	IC_50_ (mg/mL)	ACE Inhibitory Activity (%) 1.5 mg/mL
Unfractionated	1.59 ± 0.08 ^a^	48.72 ± 1.13 ^a^
MW < 3 kDa	1.14 ± 0.11 ^b^	53.01 ± 0.85 ^a^
3 kDa < MW < 10 kDa	2.19 ± 0.08 ^c^	44.16 ± 0.85 ^b^
MW > 10 kDa	2.53 ± 0.17 ^d^	34.76 ± 0.85 ^c^

Values are presented as mean ± standard deviations from triplicates (*n* = 3). Means with different lower case letters are significantly different (*p* < 0.05).

**Table 5:** Peptides identified in fraction C2-8.

Amino Acid Sequence Analysis	Mass	*m*/*z*	*z*	Area	ALC (%)	IC_50_ (μM)
FGMPLDR	834.41	418.2096	2	1.69 × 10^9^	99	219.35
MELVLR	759.43	380.7226	2	3.48 × 10^8^	99	236.85

**Table 6:** Simulated gastrointestinal digestion of synthetic peptides at 0.2 mg/mL.

Enzyme	ACE Inhibitory Activity (%)	
	FGMPLDR	MELVLR
Control	50.07 ± 0.07	59.92 ± 0.04
Pesin ^a^	52.98 ± 0.19	56.84 ± 0.04
Pesin + Trypsin ^b^	51.32 ± 0.02	58.63 ± 0.13

^a^ Pepsin hydrolysis for *2* h; ^b^ Pepsin hydrolysis for *2* h followed by trypsin hydrolysis for *2* h.
